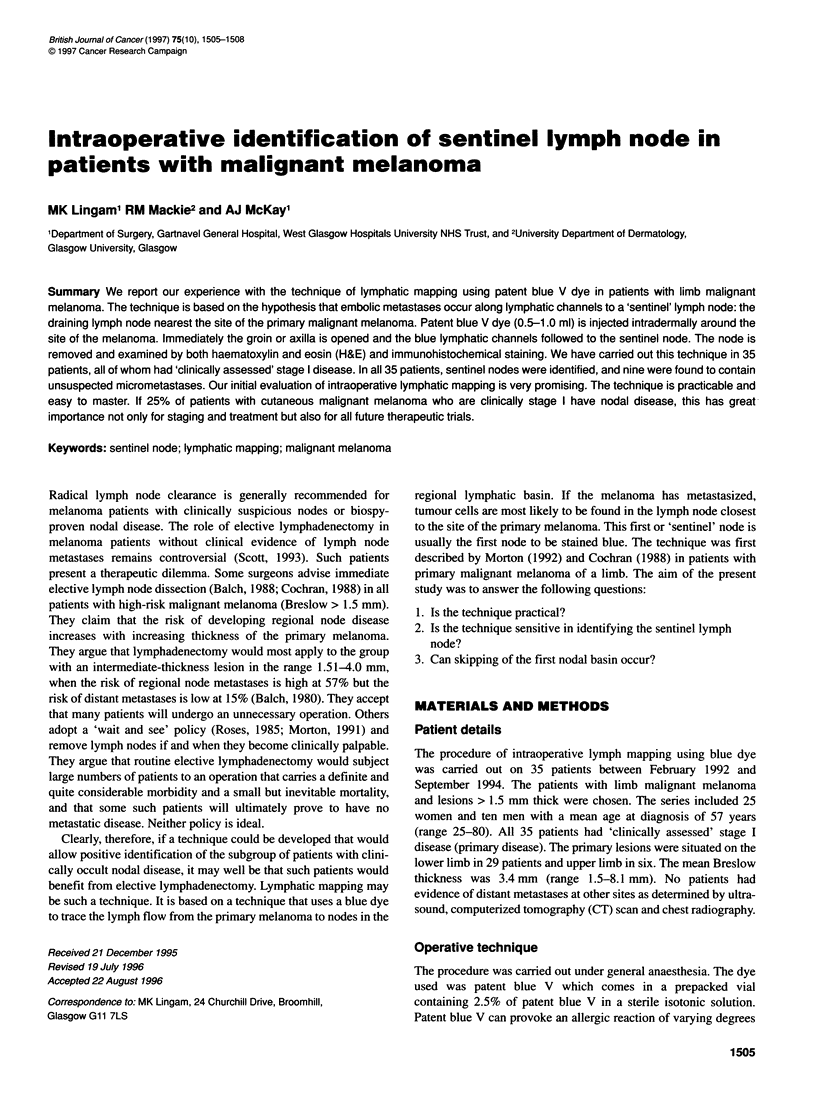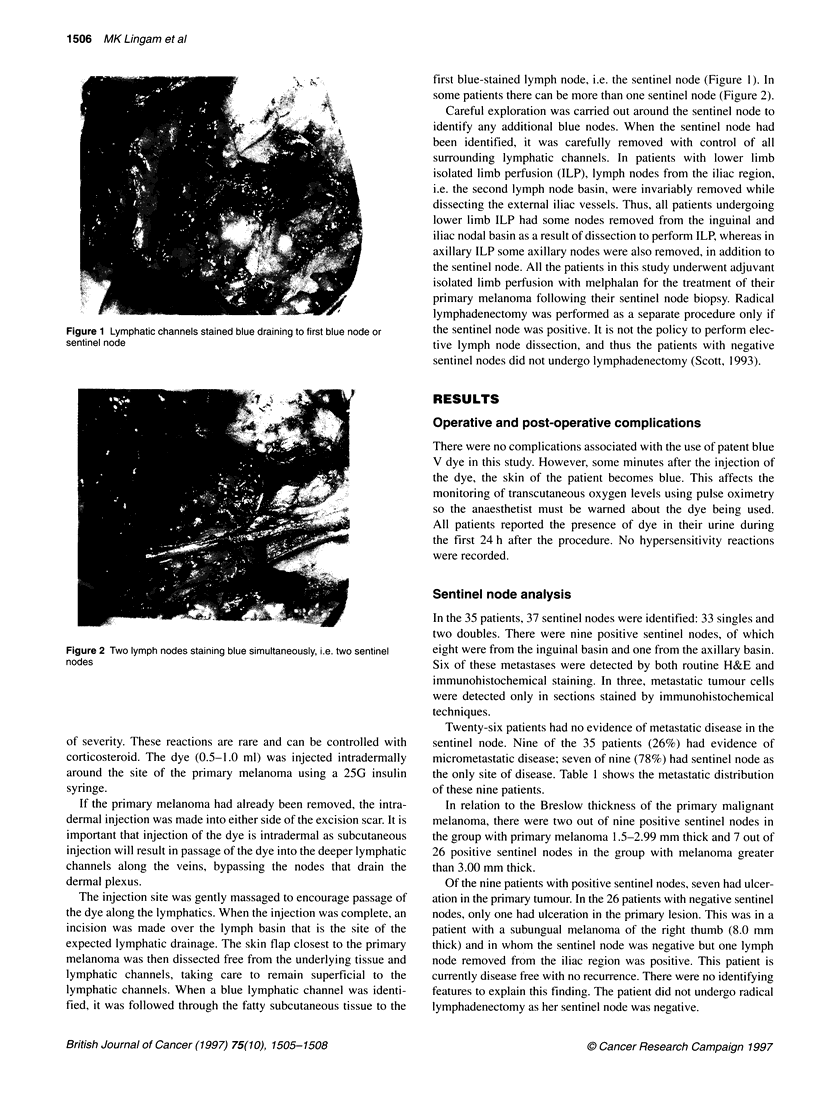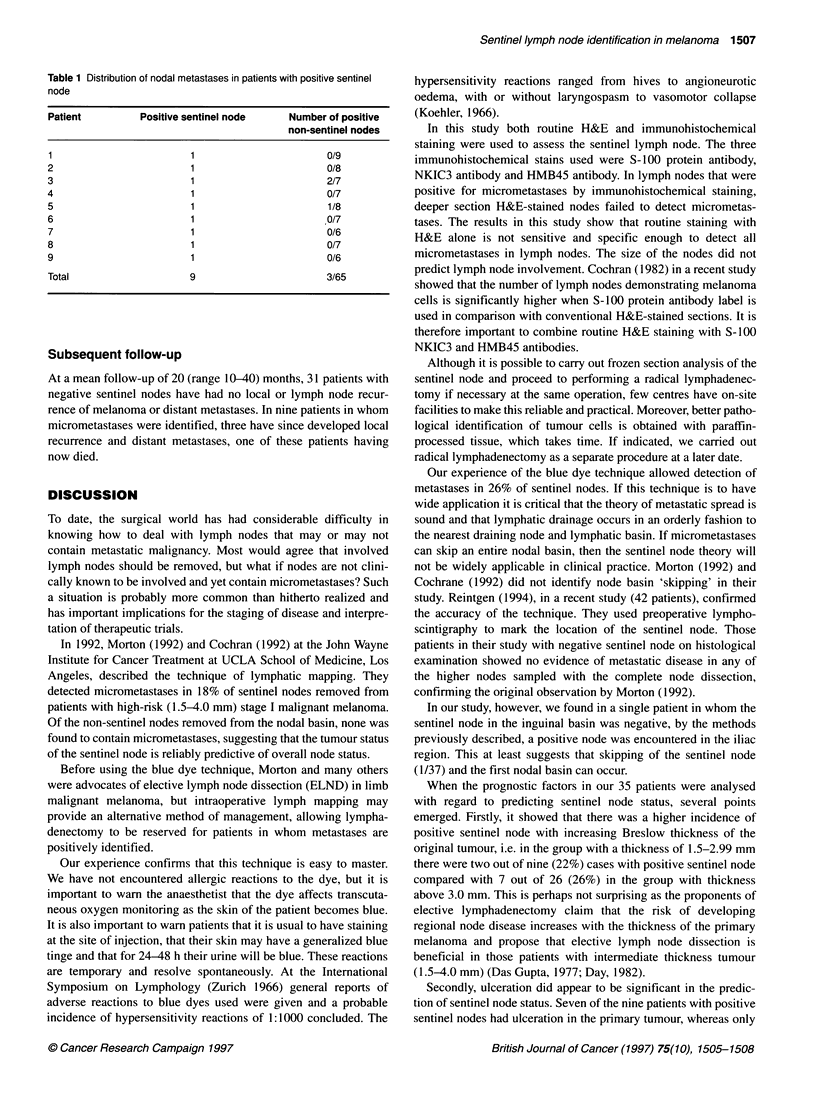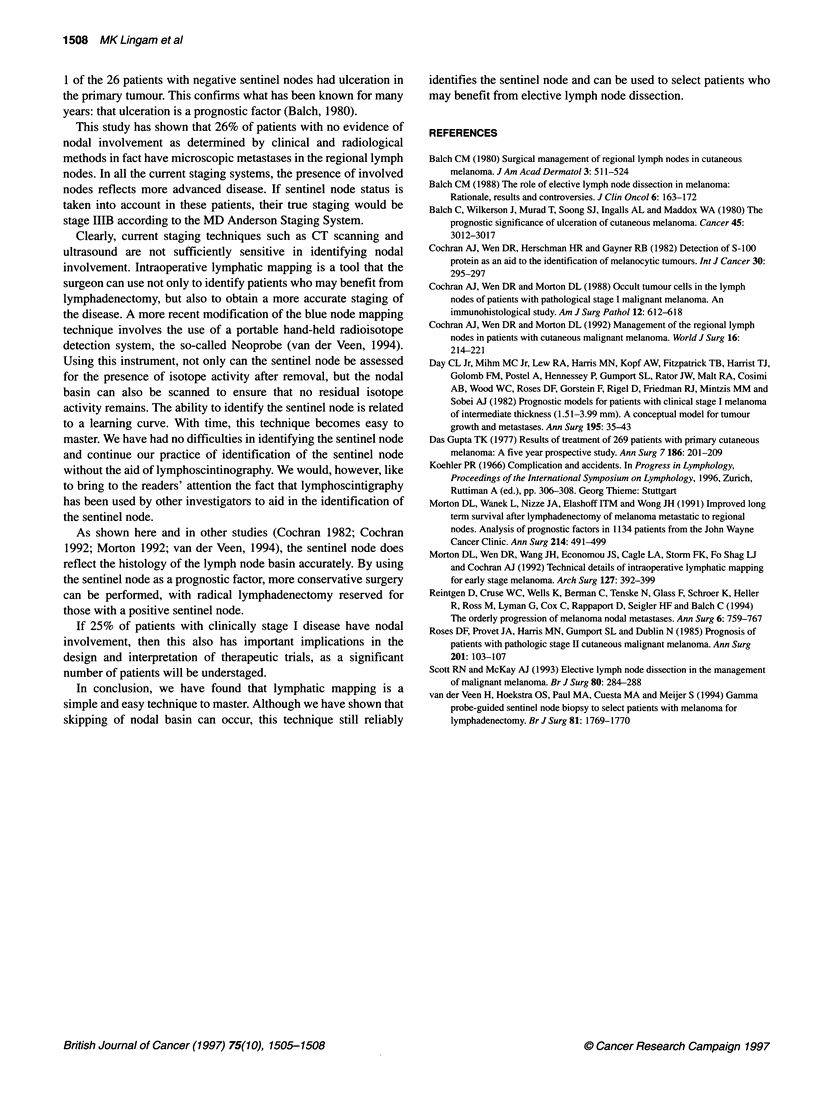# Intraoperative identification of sentinel lymph node in patients with malignant melanoma.

**DOI:** 10.1038/bjc.1997.257

**Published:** 1997

**Authors:** M. K. Lingam, R. M. Mackie, A. J. McKay

**Affiliations:** Department of Surgery, Gartnavel General Hospital, West Glasgow Hospitals University NHS Trust.

## Abstract

**Images:**


*British Journal of Cancer* (1997) **75**(10), 1505–1508
© 1997 Cancer Research Campaign

### Intraoperative identification of sentinel lymph node in patients with malignant melanoma

**MK Lingam[1] RM Mackie[2] and AJ McKay[1]**

[1]Department of Surgery, Gartnavel General Hospital, West Glasgow Hospitals University NHS Trust, and [2]University Department of Dermatology, Glasgow University, Glasgow

**Summary** We report our experience with the technique of lymphatic mapping using patent blue V dye in patients with limb malignant melanoma. The technique is based on the hypothesis that embolic metastases occur along lymphatic channels to a 'sentinel' lymph node: the draining lymph node nearest the site of the primary malignant melanoma. Patent blue V dye (0.5–1.0 ml) is injected intradermally around the site of the melanoma. Immediately the groin or axilla is opened and the blue lymphatic channels followed to the sentinel node. The node is removed and examined by both haematoxylin and eosin (H&E) and immunohistochemical staining. We have carried out this technique in 35 patients, all of whom had 'clinically assessed' stage I disease. In all 35 patients, sentinel nodes were identified, and nine were found to contain unsuspected micrometastases. Our initial evaluation of intraoperative lymphatic mapping is very promising. The technique is practicable and easy to master. If 25% of patients with cutaneous malignant melanoma who are clinically stage I have nodal disease, this has great importance not only for staging and treatment but also for all future therapeutic trials.

**Keywords:** sentinel node; lymphatic mapping; malignant melanoma

Radical lymph node clearance is generally recommended for melanoma patients with clinically suspicious nodes or biospy-proven nodal disease. The role of elective lymphadenectomy in melanoma patients without clinical evidence of lymph node metastases remains controversial (Scott, 1993). Such patients present a therapeutic dilemma. Some surgeons advise immediate elective lymph node dissection (Balch, 1988; Cochran, 1988) in all patients with high-risk malignant melanoma (Breslow > 1.5 mm). They claim that the risk of developing regional node disease increases with increasing thickness of the primary melanoma. They argue that lymphadenectomy would most apply to the group with an intermediate-thickness lesion in the range 1.51–4.0 mm, when the risk of regional node metastases is high at 57% but the risk of distant metastases is low at 15% (Balch, 1980). They accept that many patients will undergo an unnecessary operation. Others adopt a 'wait and see' policy (Roses, 1985; Morton, 1991) and remove lymph nodes if and when they become clinically palpable. They argue that routine elective lymphadenectomy would subject large numbers of patients to an operation that carries a definite and quite considerable morbidity and a small but inevitable mortality, and that some such patients will ultimately prove to have no metastatic disease. Neither policy is ideal.

Clearly, therefore, if a technique could be developed that would allow positive identification of the subgroup of patients with clinically occult nodal disease, it may well be that such patients would benefit from elective lymphadenectomy. Lymphatic mapping may be such a technique. It is based on a technique that uses a blue dye to trace the lymph flow from the primary melanoma to nodes in the regional lymphatic basin. If the melanoma has metastasized, tumour cells are most likely to be found in the lymph node closest to the site of the primary melanoma. This first or 'sentinel' node is usually the first node to be stained blue. The technique was first described by Morton (1992) and Cochran (1988) in patients with primary malignant melanoma of a limb. The aim of the present study was to answer the following questions:

1. Is the technique practical?
2. Is the technique sensitive in identifying the sentinel lymph node?
3. Can skipping of the first nodal basin occur?

### MATERIALS AND METHODS

### Patient details

The procedure of intraoperative lymph mapping using blue dye was carried out on 35 patients between February 1992 and September 1994. The patients with limb malignant melanoma and lesions > 1.5 mm thick were chosen. The series included 25 women and ten men with a mean age at diagnosis of 57 years (range 25–80). All 35 patients had 'clinically assessed' stage I disease (primary disease). The primary lesions were situated on the lower limb in 29 patients and upper limb in six. The mean Breslow thickness was 3.4 mm (range 1.5–8.1 mm). No patients had evidence of distant metastases at other sites as determined by ultrasound, computerized tomography (CT) scan and chest radiography.

### Operative technique

The procedure was carried out under general anaesthesia. The dye used was patent blue V which comes in a prepacked vial containing 2.5% of patent blue V in a sterile isotonic solution. Patent blue V can provoke an allergic reaction of varying degrees

*Received 21 December 1995*
*Revised 19 July 1996*
*Accepted 22 August 1996*

*Correspondence to:* MK Lingam, 24 Churchill Drive, Broomhill, Glasgow G11 7LS

**Figure 1** Lymphatic channels stained blue draining to first blue node or sentinel node

**Figure 2** Two lymph nodes staining blue simultaneously, i.e. two sentinel nodes of severity. These reactions are rare and can be controlled with corticosteroid. The dye (0.5–1.0 ml) was injected intradermally around the site of the primary melanoma using a 25G insulin syringe.

If the primary melanoma had already been removed, the intradermal injection was made into either side of the excision scar. It is important that injection of the dye is intradermal as subcutaneous injection will result in passage of the dye into the deeper lymphatic channels along the veins, bypassing the nodes that drain the dermal plexus.

The injection site was gently massaged to encourage passage of the dye along the lymphatics. When the injection was complete, an incision was made over the lymph basin that is the site of the expected lymphatic drainage. The skin flap closest to the primary melanoma was then dissected free from the underlying tissue and lymphatic channels, taking care to remain superficial to the lymphatic channels. When a blue lymphatic channel was identified, it was followed through the fatty subcutaneous tissue to the first blue-stained lymph node, i.e. the sentinel node (Figure 1). In some patients there can be more than one sentinel node (Figure 2).

Careful exploration was carried out around the sentinel node to identify any additional blue nodes. When the sentinel node had been identified, it was carefully removed with control of all surrounding lymphatic channels. In patients with lower limb isolated limb perfusion (ILP), lymph nodes from the iliac region, i.e. the second lymph node basin, were invariably removed while dissecting the external iliac vessels. Thus, all patients undergoing lower limb ILP had some nodes removed from the inguinal and iliac nodal basin as a result of dissection to perform ILP, whereas in axillary ILP some axillary nodes were also removed, in addition to the sentinel node. All the patients in this study underwent adjuvant isolated limb perfusion with melphalan for the treatment of their primary melanoma following their sentinel node biopsy. Radical lymphadenectomy was performed as a separate procedure only if the sentinel node was positive. It is not the policy to perform elective lymph node dissection, and thus the patients with negative sentinel nodes did not undergo lymphadenectomy (Scott, 1993).

### RESULTS

### Operative and post-operative complications

There were no complications associated with the use of patent blue V dye in this study. However, some minutes after the injection of the dye, the skin of the patient becomes blue. This affects the monitoring of transcutaneous oxygen levels using pulse oximetry so the anaesthetist must be warned about the dye being used. All patients reported the presence of dye in their urine during the first 24 h after the procedure. No hypersensitivity reactions were recorded.

### Sentinel node analysis

In the 35 patients, 37 sentinel nodes were identified: 33 singles and two doubles. There were nine positive sentinel nodes, of which eight were from the inguinal basin and one from the axillary basin. Six of these metastases were detected by both routine H&E and immunohistochemical staining. In three, metastatic tumour cells were detected only in sections stained by immunohistochemical techniques.

Twenty-six patients had no evidence of metastatic disease in the sentinel node. Nine of the 35 patients (26%) had evidence of micrometastatic disease; seven of nine (78%) had sentinel node as the only site of disease. Table 1 shows the metastatic distribution of these nine patients.

In relation to the Breslow thickness of the primary malignant melanoma, there were two out of nine positive sentinel nodes in the group with primary melanoma 1.5–2.99 mm thick and 7 out of 26 positive sentinel nodes in the group with melanoma greater than 3.00 mm thick.

Of the nine patients with positive sentinel nodes, seven had ulceration in the primary tumour. In the 26 patients with negative sentinel nodes, only one had ulceration in the primary lesion. This was in a patient with a subungual melanoma of the right thumb (8.0 mm thick) and in whom the sentinel node was negative but one lymph node removed from the iliac region was positive. This patient is currently disease free with no recurrence. There were no identifying features to explain this finding. The patient did not undergo radical lymphadenectomy as her sentinel node was negative.

*© Cancer Research Campaign 1997*

**Table 1** Distribution of nodal metastases in patients with positive sentinel node

| Patient | Positive sentinel node | Number of positive non-sentinel nodes |
|---|---|---|
| 1 | 1 | 0/9 |
| 2 | 1 | 0/8 |
| 3 | 1 | 2/7 |
| 4 | 1 | 0/7 |
| 5 | 1 | 1/8 |
| 6 | 1 | .0/7 |
| 7 | 1 | 0/6 |
| 8 | 1 | 0/7 |
| 9 | 1 | 0/6 |
| Total | 9 | 3/65 |

### Subsequent follow-up

At a mean follow-up of 20 (range 10–40) months, 31 patients with negative sentinel nodes have had no local or lymph node recurrence of melanoma or distant metastases. In nine patients in whom micrometastases were identified, three have since developed local recurrence and distant metastases, one of these patients having now died.

### DISCUSSION

To date, the surgical world has had considerable difficulty in knowing how to deal with lymph nodes that may or may not contain metastatic malignancy. Most would agree that involved lymph nodes should be removed, but what if nodes are not clinically known to be involved and yet contain micrometastases? Such a situation is probably more common than hitherto realized and has important implications for the staging of disease and interpretation of therapeutic trials.

In 1992, Morton (1992) and Cochran (1992) at the John Wayne Institute for Cancer Treatment at UCLA School of Medicine, Los Angeles, described the technique of lymphatic mapping. They detected micrometastases in 18% of sentinel nodes removed from patients with high-risk (1.5–4.0 mm) stage I malignant melanoma. Of the non-sentinel nodes removed from the nodal basin, none was found to contain micrometastases, suggesting that the tumour status of the sentinel node is reliably predictive of overall node status.

Before using the blue dye technique, Morton and many others were advocates of elective lymph node dissection (ELND) in limb malignant melanoma, but intraoperative lymph mapping may provide an alternative method of management, allowing lymphadenectomy to be reserved for patients in whom metastases are positively identified.

Our experience confirms that this technique is easy to master. We have not encountered allergic reactions to the dye, but it is important to warn the anaesthetist that the dye affects transcutaneous oxygen monitoring as the skin of the patient becomes blue. It is also important to warn patients that it is usual to have staining at the site of injection, that their skin may have a generalized blue tinge and that for 24–48 h their urine will be blue. These reactions are temporary and resolve spontaneously. At the International Symposium on Lymphology (Zurich 1966) general reports of adverse reactions to blue dyes used were given and a probable incidence of hypersensitivity reactions of 1:1000 concluded. The hypersensitivity reactions ranged from hives to angioneurotic oedema, with or without laryngospasm to vasomotor collapse (Koehler, 1966).

In this study both routine H&E and immunohistochemical staining were used to assess the sentinel lymph node. The three immunohistochemical stains used were S-100 protein antibody, NKIC3 antibody and HMB45 antibody. In lymph nodes that were positive for micrometastases by immunohistochemical staining, deeper section H&E-stained nodes failed to detect micrometastases. The results in this study show that routine staining with H&E alone is not sensitive and specific enough to detect all micrometastases in lymph nodes. The size of the nodes did not predict lymph node involvement. Cochran (1982) in a recent study showed that the number of lymph nodes demonstrating melanoma cells is significantly higher when S-100 protein antibody label is used in comparison with conventional H&E-stained sections. It is therefore important to combine routine H&E staining with S-100 NKIC3 and HMB45 antibodies.

Although it is possible to carry out frozen section analysis of the sentinel node and proceed to performing a radical lymphadenectomy if necessary at the same operation, few centres have on-site facilities to make this reliable and practical. Moreover, better pathological identification of tumour cells is obtained with paraffin-processed tissue, which takes time. If indicated, we carried out radical lymphadenectomy as a separate procedure at a later date.

Our experience of the blue dye technique allowed detection of metastases in 26% of sentinel nodes. If this technique is to have wide application it is critical that the theory of metastatic spread is sound and that lymphatic drainage occurs in an orderly fashion to the nearest draining node and lymphatic basin. If micrometastases can skip an entire nodal basin, then the sentinel node theory will not be widely applicable in clinical practice. Morton (1992) and Cochrane (1992) did not identify node basin 'skipping' in their study. Reintgen (1994), in a recent study (42 patients), confirmed the accuracy of the technique. They used preoperative lymphoscintigraphy to mark the location of the sentinel node. Those patients in their study with negative sentinel node on histological examination showed no evidence of metastatic disease in any of the higher nodes sampled with the complete node dissection, confirming the original observation by Morton (1992).

In our study, however, we found in a single patient in whom the sentinel node in the inguinal basin was negative, by the methods previously described, a positive node was encountered in the iliac region. This at least suggests that skipping of the sentinel node (1/37) and the first nodal basin can occur.

When the prognostic factors in our 35 patients were analysed with regard to predicting sentinel node status, several points emerged. Firstly, it showed that there was a higher incidence of positive sentinel node with increasing Breslow thickness of the original tumour, i.e. in the group with a thickness of 1.5–2.99 mm there were two out of nine (22%) cases with positive sentinel node compared with 7 out of 26 (26%) in the group with thickness above 3.0 mm. This is perhaps not surprising as the proponents of elective lymphadenectomy claim that the risk of developing regional node disease increases with the thickness of the primary melanoma and propose that elective lymph node dissection is beneficial in those patients with intermediate thickness tumour (1.5–4.0 mm) (Das Gupta, 1977; Day, 1982).

Secondly, ulceration did appear to be significant in the prediction of sentinel node status. Seven of the nine patients with positive sentinel nodes had ulceration in the primary tumour, whereas only

*© Cancer Research Campaign 1997*

1 of the 26 patients with negative sentinel nodes had ulceration in the primary tumour. This confirms what has been known for many years: that ulceration is a prognostic factor (Balch, 1980).

This study has shown that 26% of patients with no evidence of nodal involvement as determined by clinical and radiological methods in fact have microscopic metastases in the regional lymph nodes. In all the current staging systems, the presence of involved nodes reflects more advanced disease. If sentinel node status is taken into account in these patients, their true staging would be stage IIIB according to the MD Anderson Staging System.

Clearly, current staging techniques such as CT scanning and ultrasound are not sufficiently sensitive in identifying nodal involvement. Intraoperative lymphatic mapping is a tool that the surgeon can use not only to identify patients who may benefit from lymphadenectomy, but also to obtain a more accurate staging of the disease. A more recent modification of the blue node mapping technique involves the use of a portable hand-held radioisotope detection system, the so-called Neoprobe (van der Veen, 1994). Using this instrument, not only can the sentinel node be assessed for the presence of isotope activity after removal, but the nodal basin can also be scanned to ensure that no residual isotope activity remains. The ability to identify the sentinel node is related to a learning curve. With time, this technique becomes easy to master. We have had no difficulties in identifying the sentinel node and continue our practice of identification of the sentinel node without the aid of lymphoscintinography. We would, however, like to bring to the readers' attention the fact that lymphoscintigraphy has been used by other investigators to aid in the identification of the sentinel node.

As shown here and in other studies (Cochran 1982; Cochran 1992; Morton 1992; van der Veen, 1994), the sentinel node does reflect the histology of the lymph node basin accurately. By using the sentinel node as a prognostic factor, more conservative surgery can be performed, with radical lymphadenectomy reserved for those with a positive sentinel node.

If 25% of patients with clinically stage I disease have nodal involvement, then this also has important implications in the design and interpretation of therapeutic trials, as a significant number of patients will be understaged.

In conclusion, we have found that lymphatic mapping is a simple and easy technique to master. Although we have shown that skipping of nodal basin can occur, this technique still reliably identifies the sentinel node and can be used to select patients who may benefit from elective lymph node dissection.

### REFERENCES

Balch CM (1980) Surgical management of regional lymph nodes in cutaneous melanoma. *J Am Acad Dermatol* **3**: 511–524

Balch CM (1988) The role of elective lymph node dissection in melanoma: Rationale, results and controversies. *J Clin Oncol* **6**: 163–172

Balch C, Wilkerson J, Murad T, Soong SJ, Ingalls AL and Maddox WA (1980) The prognostic significance of ulceration of cutaneous melanoma. *Cancer* **45**: 3012-3017

Cochran AJ, Wen DR, Herschman HR and Gayner RB (1982) Detection of S-100 protein as an aid to the identification of melanocytic tumours. *Int J Cancer* **30**: 295–297

Cochran AJ, Wen DR and Morton DL (1988) Occult tumour cells in the lymph nodes of patients with pathological stage I malignant melanoma. An immunohistological study. *Am J Surg Pathol* **12**: 612–618

Cochran AJ, Wen DR and Morton DL (1992) Management of the regional lymph nodes in patients with cutaneous malignant melanoma. *World J Surg* **16**: 214–221

Day CL Jr, Mihm MC Jr, Lew RA, Harris MN, Kopf AW, Fitzpatrick TB, Harrist TJ, Golomb FM, Postel A, Hennessey P, Gumport SL, Rator JW, Malt RA, Cosimi AB, Wood WC, Roses DF, Gorstein F, Rigel D, Friedman RJ, Mintzis MM and Sobei AJ (1982) Prognostic models for patients with clinical stage I melanoma of intermediate thickness (1.51–3.99 mm). A conceptual model for tumour growth and metastases. *Ann Surg* **195**: 35–43

Das Gupta TK (1977) Results of treatment of 269 patients with primary cutaneous melanoma: A five year prospective study. *Ann Surg 7* **186**: 201–209

Koehler PR (1966) Complication and accidents. In *Progress in Lymphology, Proceedings of the International Symposium on Lymphology*, 1996, Zurich, Ruttiman A (ed.), pp. 306–308. Georg Thieme: Stuttgart

Morton DL, Wanek L, Nizze JA, Elashoff ITM and Wong JH (1991) Improved long term survival after lymphadenectomy of melanoma metastatic to regional nodes. Analysis of prognostic factors in 1134 patients from the John Wayne Cancer Clinic. *Ann Surg* **214**: 491–499

Morton DL, Wen DR, Wang JH, Economou JS, Cagle LA, Storm FK, Fo Shag LJ and Cochran AJ (1992) Technical details of intraoperative lymphatic mapping for early stage melanoma. *Arch Surg* **127**: 392–399

Reintgen D, Cruse WC, Wells K, Berman C, Tenske N, Glass F, Schroer K, Heller R, Ross M, Lyman G, Cox C, Rappaport D, Seigler HF and Balch C (1994) The orderly progression of melanoma nodal metastases. *Ann Surg* **6**: 759–767

Roses DF, Provet JA, Harris MN, Gumport SL and Dublin N (1985) Prognosis of patients with pathologic stage II cutaneous malignant melanoma. *Ann Surg* **201**: 103–107

Scott RN and McKay AJ (1993) Elective lymph node dissection in the management of malignant melanoma. *Br J Surg* **80**: 284–288

van der Veen H, Hoekstra OS, Paul MA, Cuesta MA and Meijer S (1994) Gamma probe-guided sentinel node biopsy to select patients with melanoma for lymphadenectomy. *Br J Surg* **81**: 1769–1770

© Cancer Research Campaign 1997